# 2-(4-Methyl­phen­yl)acetohydrazide

**DOI:** 10.1107/S160053681204799X

**Published:** 2012-11-28

**Authors:** A.S. Praveen, Jerry P. Jasinski, Shannon T. Krauss, H. S. Yathirajan, B. Narayana

**Affiliations:** a Department of Studies in Chemistry, University of Mysore, Manasagangotri, Mysore 570 006, India; b Department of Chemistry, Keene State College, 229 Main Street, Keene, NH 03435-2001, USA; c Department of Studies in Chemistry, Mangalore University, Mangalagangotri 574 199, India

## Abstract

In the title compound, C_9_H_12_N_2_O, the dihedral angle between the benzene ring and the mean plane of the acetohydrazide group is 88.2 (7)°. In the crystal, N—H⋯O hydrogen bonds and weak C—H⋯O inter­actions link the mol­ecules into infinite ribbons along [001].

## Related literature

For hydrazides as precursors in the synthesis of heterocyclic systems, see: Narayana *et al.* (2005 ▶). For related structures, see: Hanif *et al.* (2007 ▶); Liu & Gao (2012 ▶); Fun *et al.* (2011 ▶). For standard bond lengths, see: Allen *et al.* (1987) ▶.
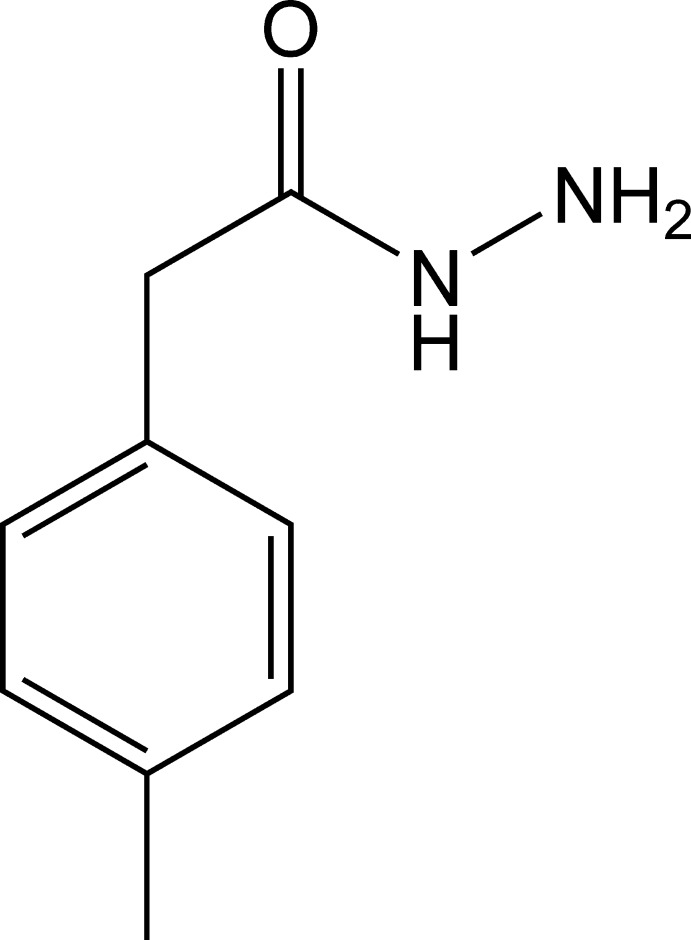


## Experimental

### 

#### Crystal data

C_9_H_12_N_2_O
*M* *_r_* = 164.21
Monoclinic, 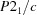

*a* = 15.4261 (16) Å
*b* = 6.2618 (7) Å
*c* = 9.2073 (10) Å
β = 106.651 (12)°
*V* = 852.09 (16) Å^3^
*Z* = 4
Cu *K*α radiation
μ = 0.69 mm^−1^
*T* = 173 K
0.32 × 0.22 × 0.08 mm

#### Data collection

Agilent Xcalibur (Eos, Gemini) diffractometer
Absorption correction: multi-scan (*CrysAlis RED*; Agilent, 2012 ▶) *T*_min_ = 0.746, *T*_max_ = 1.000
4845 measured reflections
1675 independent reflections
1359 reflections with *I* > 2σ(*I*)
*R*_int_ = 0.028

#### Refinement

*R*[*F* ^2^ > 2σ(*F* ^2^)] = 0.050
*wR*(*F* ^2^) = 0.151
*S* = 1.07
1675 reflections
119 parameters
3 restraints
H atoms treated by a mixture of independent and constrained refinement
Δρ_max_ = 0.26 e Å^−3^
Δρ_min_ = −0.21 e Å^−3^

### 

Data collection: *CrysAlis PRO* (Agilent, 2012 ▶); cell refinement: *CrysAlis PRO*; data reduction: *CrysAlis RED* (Agilent, 2012 ▶); program(s) used to solve structure: *SHELXS97* (Sheldrick, 2008 ▶); program(s) used to refine structure: *SHELXL97* (Sheldrick, 2008 ▶); molecular graphics: *SHELXTL* (Sheldrick, 2008 ▶); software used to prepare material for publication: *SHELXTL*.

## Supplementary Material

Crystal structure: contains datablock(s) global, I. DOI: 10.1107/S160053681204799X/ng5307sup1.cif

Structure factors: contains datablock(s) I. DOI: 10.1107/S160053681204799X/ng5307Isup2.hkl

Supplementary material file. DOI: 10.1107/S160053681204799X/ng5307Isup3.cml

Additional supplementary materials:  crystallographic information; 3D view; checkCIF report

## Figures and Tables

**Table 1:** Hydrogen-bond geometry (Å, °)

*D*—H⋯*A*	*D*—H	H⋯*A*	*D*⋯*A*	*D*—H⋯*A*
N2—H2⋯O1^i^	0.84 (2)	2.05 (2)	2.884 (2)	171 (2)
C2—H2*A*⋯O1^i^	0.97	2.56	3.408 (2)	146
N1—H1*A*⋯O1^ii^	0.89 (2)	2.16 (2)	3.007 (2)	159 (2)

Symmetry codes: (i) 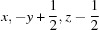; (ii) 
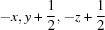
.

**Fig. 1.**
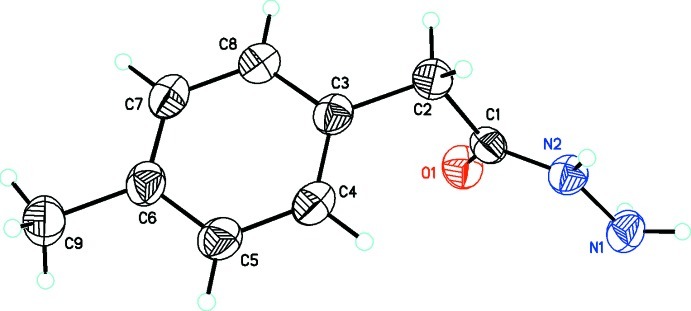
Molecular structure of the title compound showing the atom labeling scheme and 50% probability displacement ellipsoids.

**Fig. 2.**
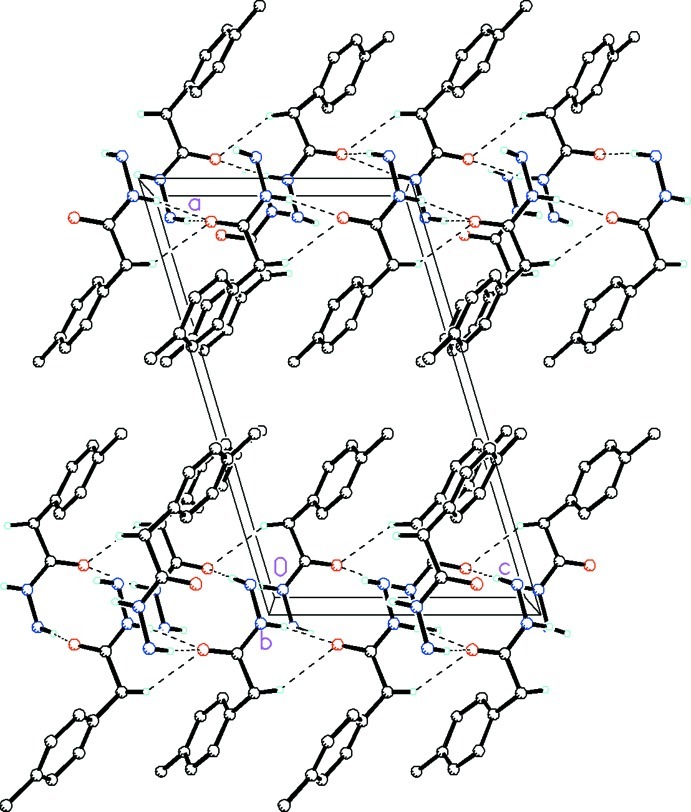
Packing diagram of the title compound viewed along the b axis. Dashed lines indicate N—H···O hydrogen bonds and weak C—H···O intermolecular interactions linking molecules into infinite 1-D chains along [001]. The remaining H atoms have been removed for clarity.

**Fig. 3.**
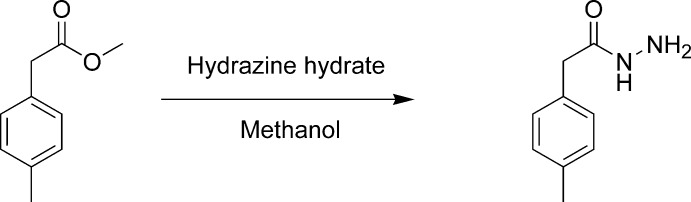
Synthesis of the title compound.

## supplementary crystallographic information

### Comment

Hydrazides are useful precursors in the synthesis of several related heterocyclic systems (Narayana *et al.*, 2005). The crystal structures of some similar hydrazides, viz., 2-(4-methoxyphenoxy)acetohydrazide (Liu & Gao, 2012), 2-(3-methoxyphenyl)acetohydrazide (Hanif *et al.*, 2007) and 2-(4-methylphenoxy)acetohydrazide (Fun *et al.*, 2011) have been reported. In view of the importance of hydrazides, the crystal structure of title compound (I) is reported.

In the title compound, C_9_H_12_N_2_O, the dihedral angle between the mean planes of the benzene ring (C3–C8) and acetohydrazide group (O1/C1/N2/N1) is 88.2 (7)° (Fig. 1). Bond lengths are in normal ranges (Allen *et al.*, 1987). In the crystal N—H···O hydrogen bonds and weak C—H···O intermolecular interactions link the molecules into infinite ribbons along [001] (Fig. 2, Table 1).

### Experimental

To a solution of methyl (4-methylphenyl)acetate (2 g, 12.18 mmol) in methanol (20 mL), hydrazine hydrate (2 mL) was added and the reaction mixture was stirred at room temperature for 6 hours (Fig. 3). After the completion of the reaction methanol was removed under vacuum, added water, precipitated solid was filtered and dried. The single crystal was grown from mixture methanol: water (2:1) by slow evaporation method and yield of the compound was 91%. (m.p.: 426-428 K).

### Refinement

H1A, H1B and H2 were restrained with DFIX = 0.86 (2)Å. All the H atoms were placed in their calculated positions and then refined using the riding model with Atom—H lengths of 0.93Å (CH), 0.97Å (CH_2_), 0.96Å (CH_3_) or 0.86Å (NH). Isotropic displacement parameters for these atoms were set to 1.19-1.21 (CH, CH_2_), 1.49 (CH_3_) or 1.20 (NH) times *U*_eq_ of the parent atom.

### Crystal data

**Table:**

C_9_H_12_N_2_O	*F*(000) = 352
*M**_r_* = 164.21	*D*_x_ = 1.280 Mg m^−^^3^
Monoclinic, *P*2_1_/*c*	Cu *K*α radiation, λ = 1.54184 Å
Hall symbol: -P 2ybc	Cell parameters from 1566 reflections
*a* = 15.4261 (16) Å	θ = 3.0–72.3°
*b* = 6.2618 (7) Å	µ = 0.69 mm^−^^1^
*c* = 9.2073 (10) Å	*T* = 173 K
β = 106.651 (12)°	Chunk, colorless
*V* = 852.09 (16) Å^3^	0.32 × 0.22 × 0.08 mm
*Z* = 4	

### Data collection

**Table:**

Agilent Xcalibur (Eos, Gemini) diffractometer	1675 independent reflections
Radiation source: Enhance (Cu) X-ray Source	1359 reflections with *I* > 2σ(*I*)
Graphite monochromator	*R*_int_ = 0.028
Detector resolution: 16.0416 pixels mm^-1^	θ_max_ = 72.5°, θ_min_ = 3.0°
ω scans	*h* = −18→19
Absorption correction: multi-scan (*CrysAlis RED*; Agilent, 2012)	*k* = −7→4
*T*_min_ = 0.746, *T*_max_ = 1.000	*l* = −10→11
4845 measured reflections	

### Refinement

**Table:**

Refinement on *F*^2^	Primary atom site location: structure-invariant direct methods
Least-squares matrix: full	Secondary atom site location: difference Fourier map
*R*[*F*^2^ > 2σ(*F*^2^)] = 0.050	Hydrogen site location: inferred from neighbouring sites
*wR*(*F*^2^) = 0.151	H atoms treated by a mixture of independent and constrained refinement
*S* = 1.07	*w* = 1/[σ^2^(*F*_o_^2^) + (0.0882*P*)^2^ + 0.1271*P*] where *P* = (*F*_o_^2^ + 2*F*_c_^2^)/3
1675 reflections	(Δ/σ)_max_ < 0.001
119 parameters	Δρ_max_ = 0.26 e Å^−^^3^
3 restraints	Δρ_min_ = −0.21 e Å^−^^3^

### Special details

**Table:**

Geometry. All esds (except the esd in the dihedral angle between two l.s. planes) are estimated using the full covariance matrix. The cell esds are taken into account individually in the estimation of esds in distances, angles and torsion angles; correlations between esds in cell parameters are only used when they are defined by crystal symmetry. An approximate (isotropic) treatment of cell esds is used for estimating esds involving l.s. planes.
Refinement. Refinement of *F*^2^ against ALL reflections. The weighted *R*-factor *wR* and goodness of fit *S* are based on *F*^2^, conventional *R*-factors *R* are based on *F*, with *F* set to zero for negative *F*^2^. The threshold expression of *F*^2^ > σ(*F*^2^) is used only for calculating *R*-factors(gt) *etc*. and is not relevant to the choice of reflections for refinement. *R*-factors based on *F*^2^ are statistically about twice as large as those based on *F*, and *R*- factors based on ALL data will be even larger.

### Fractional atomic coordinates and isotropic or equivalent isotropic displacement parameters (Å^2^)

**Table:**

	*x*	*y*	*z*	*U*_iso_*/*U*_eq_	
O1	0.08543 (9)	0.1283 (2)	0.28834 (14)	0.0417 (4)	
N1	−0.06203 (11)	0.2284 (3)	0.0460 (2)	0.0396 (4)	
H1A	−0.0731 (15)	0.323 (3)	0.110 (2)	0.048*	
H1B	−0.0656 (15)	0.105 (3)	0.093 (2)	0.048*	
N2	0.03002 (10)	0.2537 (2)	0.05112 (18)	0.0336 (4)	
H2	0.0399 (15)	0.293 (3)	−0.030 (2)	0.040*	
C1	0.09783 (12)	0.1991 (3)	0.17011 (19)	0.0319 (4)	
C2	0.19169 (12)	0.2268 (3)	0.1528 (2)	0.0370 (4)	
H2A	0.1873	0.2917	0.0553	0.044*	
H2B	0.2199	0.0878	0.1551	0.044*	
C3	0.25012 (11)	0.3654 (3)	0.27779 (19)	0.0340 (4)	
C4	0.22379 (12)	0.5730 (3)	0.2991 (2)	0.0381 (4)	
H4	0.1707	0.6279	0.2343	0.046*	
C5	0.27565 (12)	0.6990 (3)	0.4157 (2)	0.0394 (4)	
H5	0.2562	0.8363	0.4292	0.047*	
C6	0.35650 (12)	0.6232 (3)	0.5132 (2)	0.0381 (4)	
C7	0.38281 (12)	0.4170 (3)	0.4904 (2)	0.0402 (5)	
H7	0.4366	0.3630	0.5538	0.048*	
C8	0.33043 (12)	0.2894 (3)	0.3747 (2)	0.0373 (4)	
H8	0.3495	0.1515	0.3621	0.045*	
C9	0.41413 (15)	0.7621 (4)	0.6381 (2)	0.0518 (6)	
H9A	0.3783	0.8125	0.7009	0.078*	
H9B	0.4365	0.8817	0.5944	0.078*	
H9C	0.4642	0.6802	0.6985	0.078*	

### Atomic displacement parameters (Å^2^)

**Table:**

	*U*^11^	*U*^22^	*U*^33^	*U*^12^	*U*^13^	*U*^23^
O1	0.0437 (8)	0.0512 (8)	0.0323 (7)	−0.0037 (6)	0.0144 (6)	0.0019 (5)
N1	0.0299 (8)	0.0442 (9)	0.0452 (10)	−0.0016 (6)	0.0115 (7)	−0.0051 (7)
N2	0.0311 (8)	0.0391 (8)	0.0321 (8)	−0.0006 (6)	0.0113 (6)	0.0010 (6)
C1	0.0347 (9)	0.0320 (8)	0.0298 (9)	−0.0025 (6)	0.0105 (7)	−0.0047 (6)
C2	0.0328 (9)	0.0492 (10)	0.0307 (9)	−0.0012 (7)	0.0119 (7)	−0.0038 (7)
C3	0.0293 (8)	0.0435 (10)	0.0310 (9)	−0.0022 (7)	0.0113 (7)	0.0004 (7)
C4	0.0288 (9)	0.0460 (10)	0.0392 (10)	0.0037 (7)	0.0095 (7)	0.0048 (7)
C5	0.0327 (9)	0.0409 (10)	0.0468 (11)	−0.0015 (7)	0.0151 (8)	−0.0014 (8)
C6	0.0303 (9)	0.0484 (10)	0.0374 (10)	−0.0060 (7)	0.0126 (7)	−0.0032 (8)
C7	0.0301 (9)	0.0496 (11)	0.0388 (10)	0.0012 (7)	0.0065 (7)	0.0040 (8)
C8	0.0340 (9)	0.0386 (9)	0.0398 (10)	0.0027 (7)	0.0115 (8)	0.0021 (7)
C9	0.0403 (11)	0.0650 (14)	0.0498 (12)	−0.0083 (9)	0.0121 (10)	−0.0157 (10)

### Geometric parameters (Å, º)

**Table:**

O1—C1	1.240 (2)	C4—C5	1.387 (3)
N1—N2	1.416 (2)	C4—H4	0.9300
N1—H1A	0.889 (16)	C5—C6	1.395 (3)
N1—H1B	0.898 (15)	C5—H5	0.9300
N2—C1	1.325 (2)	C6—C7	1.388 (3)
N2—H2	0.842 (15)	C6—C9	1.511 (3)
C1—C2	1.511 (2)	C7—C8	1.390 (3)
C2—C3	1.515 (2)	C7—H7	0.9300
C2—H2A	0.9700	C8—H8	0.9300
C2—H2B	0.9700	C9—H9A	0.9600
C3—C8	1.387 (2)	C9—H9B	0.9600
C3—C4	1.393 (3)	C9—H9C	0.9600
			
N2—N1—H1A	106.6 (15)	C3—C4—H4	119.5
N2—N1—H1B	106.3 (15)	C4—C5—C6	121.05 (17)
H1A—N1—H1B	102 (2)	C4—C5—H5	119.5
C1—N2—N1	123.05 (15)	C6—C5—H5	119.5
C1—N2—H2	120.6 (15)	C7—C6—C5	117.77 (17)
N1—N2—H2	116.0 (15)	C7—C6—C9	121.12 (17)
O1—C1—N2	122.32 (16)	C5—C6—C9	121.11 (18)
O1—C1—C2	121.82 (16)	C6—C7—C8	121.30 (16)
N2—C1—C2	115.86 (15)	C6—C7—H7	119.3
C1—C2—C3	111.39 (14)	C8—C7—H7	119.3
C1—C2—H2A	109.3	C3—C8—C7	120.79 (17)
C3—C2—H2A	109.3	C3—C8—H8	119.6
C1—C2—H2B	109.3	C7—C8—H8	119.6
C3—C2—H2B	109.3	C6—C9—H9A	109.5
H2A—C2—H2B	108.0	C6—C9—H9B	109.5
C8—C3—C4	118.18 (16)	H9A—C9—H9B	109.5
C8—C3—C2	121.29 (16)	C6—C9—H9C	109.5
C4—C3—C2	120.52 (15)	H9A—C9—H9C	109.5
C5—C4—C3	120.90 (16)	H9B—C9—H9C	109.5
C5—C4—H4	119.5		
			
N1—N2—C1—O1	−2.6 (3)	C3—C4—C5—C6	1.2 (3)
N1—N2—C1—C2	177.00 (15)	C4—C5—C6—C7	−0.6 (3)
O1—C1—C2—C3	−55.6 (2)	C4—C5—C6—C9	178.63 (17)
N2—C1—C2—C3	124.79 (16)	C5—C6—C7—C8	−0.1 (3)
C1—C2—C3—C8	120.96 (18)	C9—C6—C7—C8	−179.37 (18)
C1—C2—C3—C4	−58.4 (2)	C4—C3—C8—C7	0.4 (3)
C8—C3—C4—C5	−1.1 (3)	C2—C3—C8—C7	−179.03 (16)
C2—C3—C4—C5	178.30 (16)	C6—C7—C8—C3	0.3 (3)

### Hydrogen-bond geometry (Å, º)

**Table:**

*D*—H···*A*	*D*—H	H···*A*	*D*···*A*	*D*—H···*A*
N2—H2···O1^i^	0.84 (2)	2.05 (2)	2.884 (2)	171 (2)
C2—H2*A*···O1^i^	0.97	2.56	3.408 (2)	146
N1—H1*A*···O1^ii^	0.89 (2)	2.16 (2)	3.007 (2)	159 (2)

Symmetry codes: (i) *x*, −*y*+1/2, *z*−1/2; (ii) −*x*, *y*+1/2, −*z*+1/2.
